# Differential Effects of pH-Shift and Ultrasound Intervention Sequences on Physicochemical, Structural and Gelling Properties of Potato–Egg White Composite Proteins

**DOI:** 10.3390/gels12080686

**Published:** 2026-08-03

**Authors:** Jing Li, Huimin Lu, Hongxi Zhao, Qiannan Liu, Ruixuan Zhao, Honghai Hu

**Affiliations:** 1 Institute of Food Science and Technology, Chinese Academy of Agricultural Sciences, Comprehensive Key Laboratory of Agro-Products Processing, Ministry of Agriculture and Rural Affairs, Beijing 100193, China; lijing08@caas.cn (J.L.); min184andlllll@foxmail.com (H.L.); zhaohongxi0127@163.com (H.Z.); liuqnbuct@163.com (Q.L.); 2 Guangdong Provincial Key Laboratory of Aquatic Product Processing and Safety, College of Food Science and Technology, Guangdong Ocean University, Zhanjiang 524088, China; 3 State Key Laboratory of Food Nutrition and Safety, Food Biotechnology Engineering Research Center of Ministry of Education, Tianjin University of Science & Technology, Tianjin 300457, China

**Keywords:** potato protein, pH shift, ultrasound intervention sequences, structure, gelling property

## Abstract

Composited proteins from animal and plant sources can address the limitations of single proteins and expand their applications in food processing. In this study, potato protein and egg white protein (PP/EWP) composites were modified through pH-shift and ultrasound treatments. A systematic comparison was conducted, for the first time, among three ultrasound application protocols (US-A-pH, US-P-pH, and US-B-pH, corresponding to ultrasound after, during, and before pH-shift treatment, respectively) over a range of six pH conditions (3, 4, 5, 8, 9, 10) in a composite system. It was found that PP/EWP treated with alkaline pH shifts (especially pH 9 and 10) exhibited better physicochemical, structural, and gelling properties. The complexes pretreated with US-P-pH (pH = 9) possessed higher solubility, smaller particle size, enhanced hydrophobicity, and higher number of disulfide bonds. Furthermore, this treatment yielded gels with superior hardness, chewiness, adhesion, water-holding capacity, and finer network structure. US-B-pH treatment increased the random coil content of PP/EWP, producing flexible and disordered protein states and gels with higher elasticity. In contrast, US-A-pH induced significant structural changes in the protein particles, but did not yield the required gel quality. These findings were intended to offer practical guidance for designing green and efficient protein modification strategies in food processing, with promising applications in composite gel products.

## 1. Introduction

According to the United Nations’ projections, the global human population is expected to reach approximately 10.4 billion by the year 2086 [1]. The rapid population growth, coupled with rising standards of human well-being, is placing significant pressure on global protein demand [2]. In this context, alternative plant-based foods, due to their high resource utilization efficiency and low environmental impact, are of great importance in alleviating this challenge [3,4]. However, most single-source plant proteins have functional limitations, including low solubility, poor emulsifying ability, and weak gelation, which restrict their applications. To address these drawbacks, various strategies such as physical, chemical, or enzymatic modification of proteins, as well as the synergistic combination of animal and plant proteins, have emerged as effective approaches to enhance protein functionality [5,6].

Potato protein (PP) is regarded as a promising non-animal protein due to its well-balanced amino acid composition, high biological value, and low allergenicity [7]. It is considered a safe food ingredient in artificial meat formulations, and serves as a lysine supplement and a substitute for wheat products [8]. Commercially, PP is extracted from potato fruit juice (PFJ), a byproduct of the potato starch industry, which has an annual production capacity of up to 240,000 tons [9]. However, the conventional thermal coagulation method used to recover PP results in poor processing performance, including low solubility, weak emulsifying capacity, and reduced gelation ability. By contrast, egg white proteins (EWPs) are rich in sulfur-containing amino acids and are widely used in food processing due to their excellent foaming, emulsifying, and heat-induced gelling properties [10]. Theoretically, combining PP with EWP not only balances the amino acid composition but also offsets the functional limitations of each protein through intermolecular interactions between the two proteins.

Achieving ideal gel properties in complex multi-protein systems remains challenging, primarily due to differences in protein sources and sizes [11]. To improve the gel properties of proteins, various physical and chemical methods have been adopted to alter their folding states and structures. pH-shift processing is a simple yet efficient physical modification method, which involves dissolving proteins under extreme acidic or alkaline conditions to induce complete protein unfolding, followed by neutralization to achieve refolding [12]. This treatment effectively exposes hydrophobic groups and reactive sulfhydryl groups, thereby increasing protein solubility and enhancing interfacial activity. In recent years, ultrasonication, as a green processing technology, has been widely adopted to assist protein modification [13,14]. The cavitation, mechanical shear, and micro-streaming effects generated by ultrasound can disrupt protein aggregates, alter the secondary and tertiary structures of proteins, and synergistically enhance protein functionality. Recent studies have applied pH shift combined with ultrasound to protein modification, such as soy or pea isolates [12,13]. However, few studies have systematically varied the sequence of ultrasound intervention (before, during, or after pH shift) in a composite animal–plant protein system, nor have they compared acidic versus alkaline shifts across a wide pH range. The present study directly addressed these gaps by offering the first systematic evaluation of three ultrasound–pH-shift protocols on potato–egg white composite proteins.

Therefore, the primary objectives of this study were to evaluate the effects of different pH-shift conditions (acidic: pH 3, 4, 5; alkaline: pH 8, 9, 10) on the physicochemical, structural, and gelling properties of potato–egg white composite proteins, and to systematically compare three ultrasound intervention sequences applied either after, during, or before the pH shift (US-A-pH, US-P-pH, and US-B-pH, respectively). Second, we sought to identify the optimal combined treatment that maximizes solubility, gel hardness, water-holding capacity, and microstructural uniformity. The structure–function relationships underlying the observed changes were systematically investigated, particularly regarding disulfide bond formation, surface hydrophobicity, and secondary structure alterations. These findings were intended to offer practical guidance for designing green and efficient protein modification strategies in food processing, with promising applications in composite gel products.

## 2. Results and Discussion

The effects of different pH-shift conditions (acidic pH 3,4,5 vs. alkaline pH 8,9,10) and three ultrasound intervention sequences (US-A-pH, US-P-pH, US-B-pH) on the properties of PP/EWP composites were investigated. Specifically, solubility, particle size, structural characteristics (surface hydrophobicity, fluorescence, secondary structure, sulfhydryl/disulfide content, electrophoresis), and gel properties (texture, water-holding capacity, rheology, microstructure) were measured. The results are presented and discussed in the following subsections.

### 2.1. Effects of Different Modification Treatments on the Solubility and Particle Size of PP/EWP

**Figure 1:**
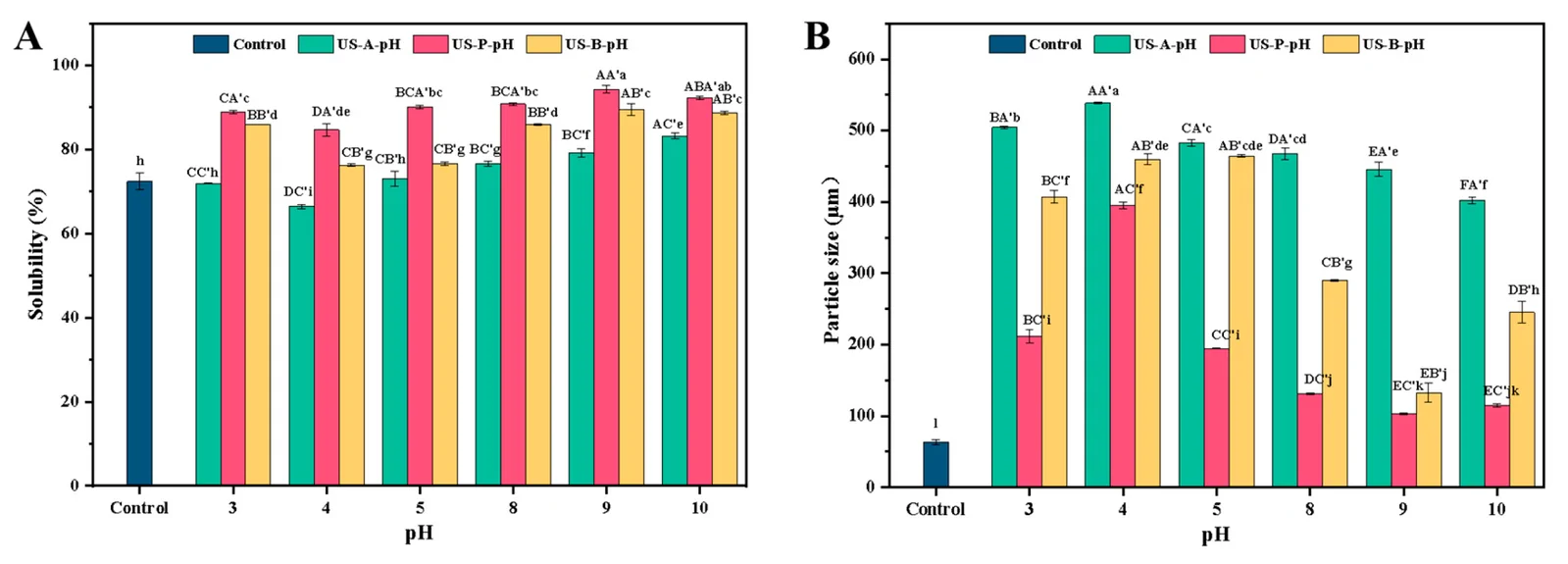
Solubility (**A**) and particle size (**B**) of PP/EWP with various ultrasound and pH-shift treatments. US-A-pH: ultrasound after pH-shift treatment; US-P-pH: ultrasound in the process of pH-shift treatment; US-B-pH: ultrasound before pH-shift treatment. Different superscript letters indicate significant differences (*p* < 0.05).

The solubility results were shown in Figure 1A. The solubility of PP/EWP prepared by alkaline pH-shift treatment (pH = 9, 10) was generally higher than of that treated with acidic pH, reaching the highest value at pH 9. Overall, samples obtained by the US-P-pH treatment (pH = 9) showed the highest solubility (94.33%) compared with the others. At alkaline pH, deprotonation of acidic amino acid residues (glutamic and aspartic acids) endows protein molecules with a higher net negative charge, thereby enhancing electrostatic repulsion and effectively suppressing aggregation—a primary driver of increased solubility. In parallel, alkaline conditions weaken hydrophobic interactions and hydrogen bonds, facilitating polypeptide chain unfolding [13]. Upon neutralization, these unfolded chains refold into more soluble conformations. Accordingly, the superior solubility observed under alkaline pH shifts, relative to acidic ones, can be attributed to the more extensive unfolding at alkaline pH, which exposes a greater number of hydrophilic residues that promote solvation during refolding. The compositional difference between the two proteins also plays a role. Since EWP contains a higher proportion of acidic amino acids (pI ≈ 4.5–5.0) than PP, it carries more net negative charge at alkaline pH, enhancing electrostatic repulsion and preventing aggregation. The superior solubility of US-P-pH suggests that cavitation disrupts nascent aggregates precisely at the point of refolding, producing smaller, more uniform particles. It should be noted that single-protein controls were not included in this study, so the synergistic versus individual effects of each protein cannot be fully deconvoluted.

The particle size of PP/EWP significantly increased after combined pH-shift and ultrasonic treatment (Figure 1B). Ultrasonication exerted cavitation forces and shear stresses that mechanically disrupted protein aggregates, while the pH-shift process induced conformational rearrangements that effectively suppressed re-aggregation, ultimately yielding smaller and more uniformly distributed particles. The increase in particle size was greater under acidic pH-shift progress (an average increase in particle size of 5.37) than under alkaline pH shift (an average increase in particle size of 3.06) (*p* < 0.05). Regarding the different processing sequences, the changes in particle size were consistent with the solubility results. The particle size of PP/EWP prepared by US-P-pH was the smallest, followed by US-B-pH and then US-A-pH. The superior solubility of US-P-pH suggested that cavitation disrupts nascent aggregation precisely at the point of refolding, producing smaller, more uniform particles. Similarly, the particle size of samples obtained by US-P-pH treatment (pH = 9) was smallest as compared with others.

### 2.2. Effects of Modification Treatments on the Structural Characteristics of PP/EWP

#### 2.2.1. Surface Hydrophobicity (H_0_) and Intrinsic Fluorescence Spectrum

**Figure 2:**
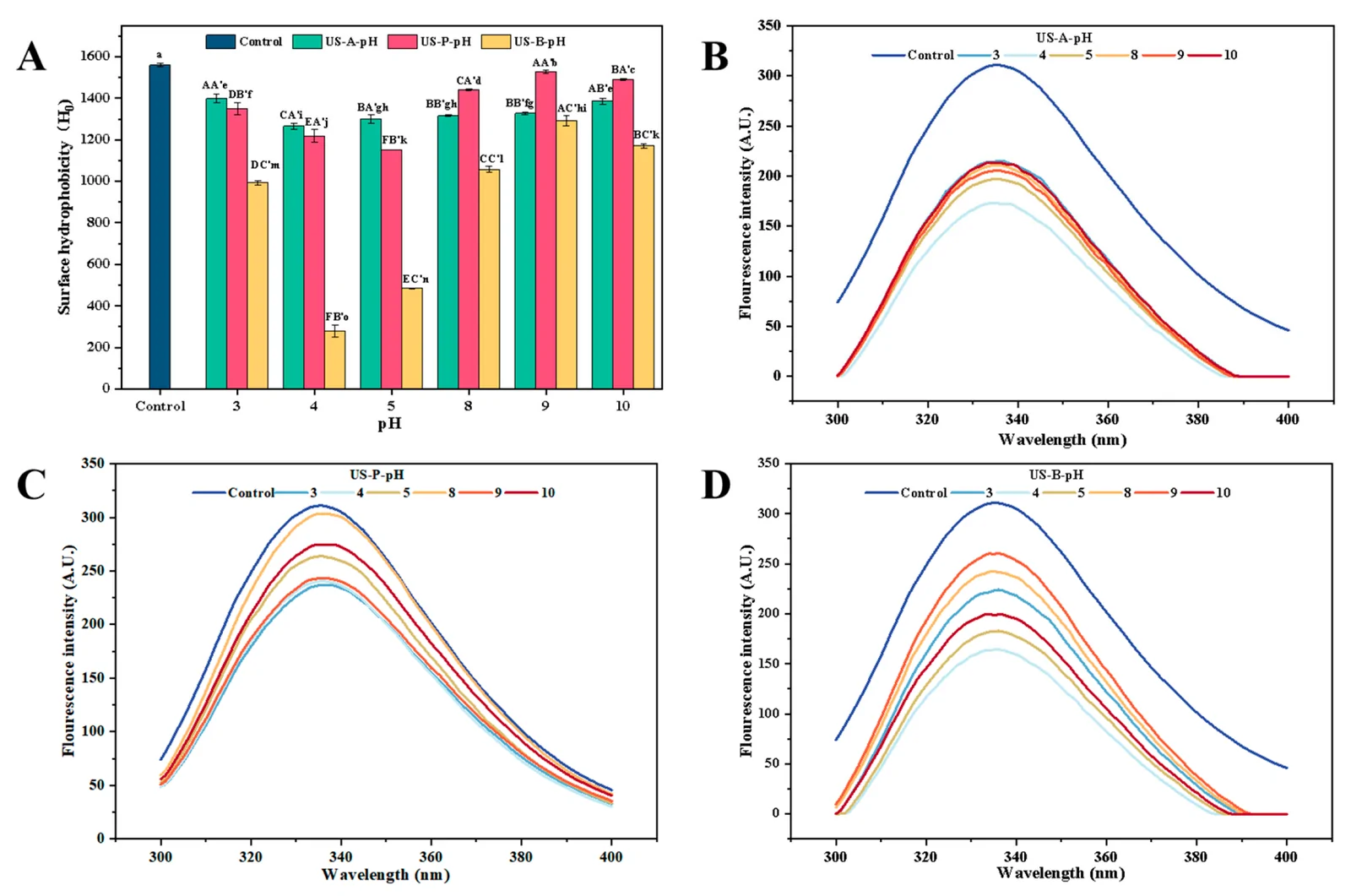
Surface hydrophobicity (**A**) of PP/EWP after various treatments and intrinsic fluorescence spectra of PP/EWP prepared by US-A-pH (**B**), US-P-pH (**C**), and US-B-pH (**D**) treatments. US-A-pH: ultrasound after pH-shift treatment; US-P-pH: ultrasound in the process of pH-shift treatment; US-B-pH: ultrasound before pH-shift treatment. Different superscript letters indicate significant differences (*p* < 0.05).

The surface hydrophobicity (H_0_) reflects the number of hydrophobic groups exposed on the protein surface, which significantly influences the tertiary and quaternary structures of the protein [15]. H_0_ was quantified experimentally using the ANS fluorescence probe (Sinopharm Chemical Reagents Co., Ltd., Beijing, China. It has been added in the Materials and Methods section), with higher slope values directly indicating greater exposure of hydrophobic patches. As shown in Figure 2A, the combined pH-shift and ultrasound treatment did not increase surface hydrophobicity relative to the control under most conditions, except for US-P-pH at pH 9 and pH 10. Under alkaline pH shifts (pH 8–10), H_0_ values were consistently higher than those recorded under acidic shifts. Among the three ultrasound sequences, US-P-pH produced the highest H_0_ at each pH level, followed by US-A-pH, whereas US-B-pH consistently yielded the lowest values. This might be due to the fact that when the mixed proteins returned to a neutral environment, the electrostatic repulsion between the proteins increased, causing them to refold, while the hydrophobic interactions weakened. The combination of alkaline shift and ultrasonic treatment caused exposure of more hydrophobic groups compared with acidic pH shift, indicating that the protein structure unfolded more in an alkaline environment and induced more protein conformational changes. Similar findings have been reported for soy protein [16]. A higher H_0_ was observed under US-P-pH treatment, and it reached its maximum value under US-P-pH (pH = 9). This implied that the cavitation effects of ultrasound, combined with the polarity of the alkali, stimulates the unfolding of protein molecules, thereby disrupting the hydrophobic interactions and causing the hydrophilic groups to be further exposed to the surrounding environment [17,18]. US-B-pH consistently exhibited the lowest H_0_ at the same pH-shift conditions, possibly because ultrasound caused the proteins to unfold, resulting in the extension of more non-covalent bonds, thereby masking the hydrophobic regions. The patatin fraction of PP (≈40% of PP) is predominantly hydrophilic, while the ovalbumin fraction of EWP (≈54% of EWP) contains a hydrophobic core. Under US-B-pH, pre-ultrasonication may preferentially fragment EWP, exposing hydrophilic peptides that subsequently mask hydrophobic regions during refolding. This compositional difference helps explain the lowest H_0_ of US-B-pH under the same pH-shift conditions.

The fluorescence spectrum of proteins is mainly related to the presence of aromatic amino acids, among which tyrosine (Tyr) and tryptophan (Trp) play key roles in determining the fluorescence intensity. This is an important method for detecting changes in the tertiary structure of proteins. The fluorescence spectra of PP/EWP are shown in Figure 2B–D. Compared with the control group, the fluorescence intensity of the PP/EWP treated with both ultrasound and pH shift decreased. Under acidic shift treatment, the fluorescence intensity of PP/EWP was lower than that under alkaline shift. The electrostatic repulsion between proteins was lower at acidic pH, which did not cause the protein structure to overly unfold, thereby reducing the number of exposed chromophores and consequently lowering the fluorescence intensity of the mixed proteins [18]. Under the same pH shift, the fluorescence intensity of PP/EWP treated with US-P-pH was greater than that of the other two treatments, possible due to the melting state of PP/EWP. The energy generated by ultrasound may enhance the melting state of the mixture returning to neutrality, resulting in a looser structure and exposing more chromophores [19,20]. Together with the results of surface hydrophobicity, it can be inferred that the US-A-pH treatment affected the tertiary structure of PP/EWP, but change in pH did not significantly impact PP/EWP.

#### 2.2.2. Protein Secondary Structure

As shown in Table 1, the percentages of α-helix, β-sheet, β-turn, and random coil of control PP/EWP were 27.88%, 40.20%, 17.07%, and 14.85%, respectively. The *α*-helix contents of US-A-pH, US-P-pH, and US-B-pH samples were reduced by 32.5–72.7%, 5.1–25.1%, and 23.4–63.6% compared to the control group, respectively. This indicated that the PP/EWP structure unfolded because *α*-helix, the most common repetitive structure in proteins, is maintained by intra-strand hydrogen bonds [21]. The disruption of the *α*-helix structure caused by US-A-pH was more severe. Under these conditions, the ‘molten globule’ structure may be more susceptible to the microfluidic force generated by ultrasound than others [22]. The differences in *α*-helix contents further indicate that the effect of ultrasound on the ‘molten globule’ structure was greater than that of the unfolded and native state composite protein. In addition, the content of *α*-helix serves as a guide for optimizing protein gelation processing [23].

The *β*-sheet levels in the PP/PWP composites prepared under the US-A-pH _pH=5,8,9_ and US-B-pH _pH=3,9_ treatments were significantly higher than those in the control group. The decline in content of *α*-helix and increase in content of *β*-sheet might result from the disruption of hydrogen bonds when samples are subjected to shear force and microfluidic force generated by ultrasound. Moreover, the increase in the content of *β*-sheet indicated that the intermolecular forces between proteins were enhanced, but the heat stability deteriorated [24].

The reduction in the content of *α*-helix was accompanied by a significant increase in random coil and *β*-turn contents. In all samples, the percentage of random coil in the US-B-pH _pH=9_ treatment group was the highest (28.54%), while the percentage of *α*-helix was the lowest (10.11%)_._ This phenomenon meant that the flexibility of proteins was increased, and protein–protein interaction may be enhanced [25]. Based on the changes in protein secondary structure content, it is hypothesized that ultrasonication of protein systems with different folding states produced composite protein particles with different degrees of looseness, resulting in different rates of protein denaturation and refolding.

#### 2.2.3. Analysis of Surface Free Sulfhydryl Groups (SH_F_) and Disulfide Bond (S-S) Contents

**Figure 3:**
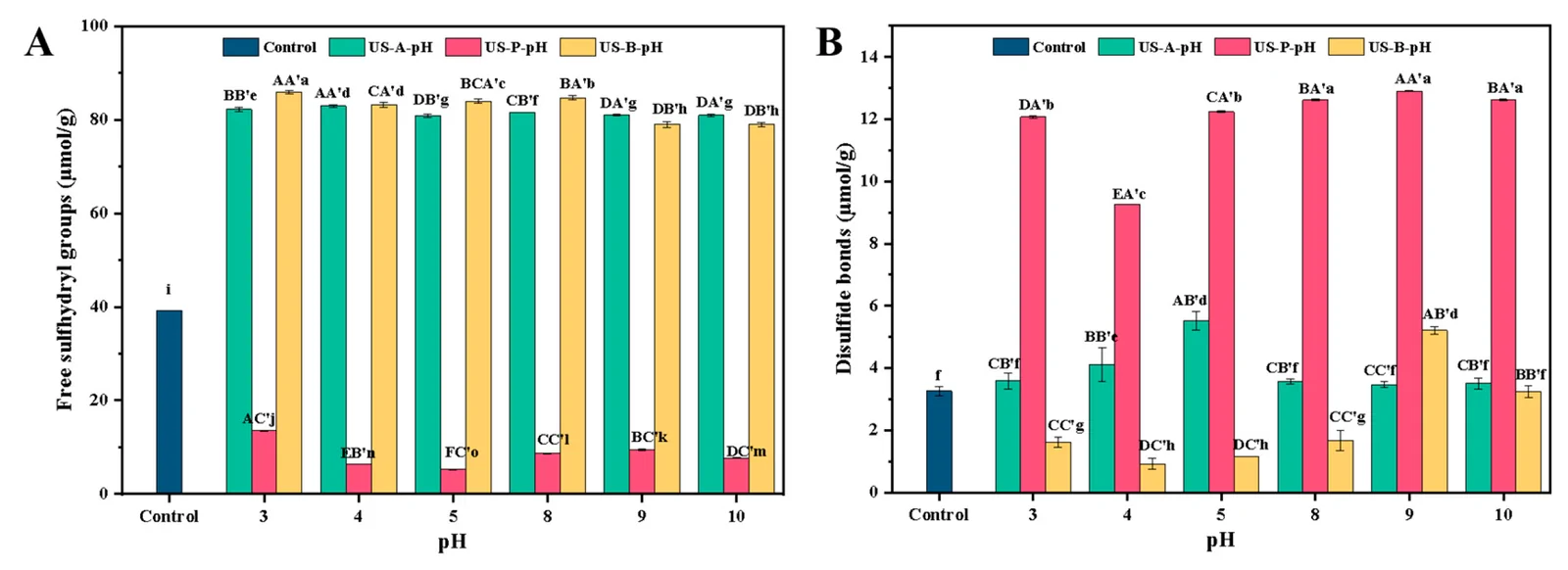
Free sulfhydryl groups (**A**) and disulfide bond contents (**B**) of PP/EWP prepared by various ultrasound and pH-shift treatments. US-A-pH: ultrasound after pH-shift treatment; US-P-pH: ultrasound in the process of pH-shift treatment; US-B-pH: ultrasound before pH-shift treatment. Different superscript letters indicate significant differences (*p* < 0.05).

Disulfide bonds, which maintain the protein structure, are formed by coupling two thiol groups. Thus, the splitting of such bonds may expose SH on the protein surface [20]. The free sulfhydryl content reflects the degree of protein structural unfolding, and there are interchange reactions between SH groups and S-S bonds [26]. The increase in disulfide bond content was quantitatively confirmed by measuring free and total sulfhydryl groups using Ellman’s reagent. As shown in Figure 3A, the SH**_F_** content significantly increased in US-A-pH and US-B-pH groups. This result can be explained as a greater degree of structural expansion, which leads to an increase in SH, as evidenced by the larger particle sizes (Figure 1B). However, in the US-A-pH and US-B-pH samples (pH = 9, 10), the S-S contents did not decrease as expected. This indicated that the protein-interaction network maintained by S-S may exist in these protein particles. Additionally, after the samples were treated under US-P-pH conditions, the SH**_F_** level decreased, which was confirmed by the increase in disulfide bond levels. This was because the exposed active sulfhydryl groups in unfolded proteins are easily oxidized by ultrasound and are buried within the protein structure during refolding, thereby leading to an increase in S-S content [13]. These thiol-blocking compounds weaken protein aggregation during thermal treatment, thereby enhancing thermal stability by inhibiting the reactions of active sulfhydryl groups [27]. Moreover, despite the existence of electrostatic repulsion during the ultrasonic treatment of US-P-pH, which reduces the disulfide bond cross-linking of microparticles, this effect is not as strong as the oxidative effect [13]. EWP contains approximately 2.3% cysteine plus cystine, whereas potato protein contains only 0.6–0.8% sulfur amino acids. The higher S-S content observed under US-P-pH treatment (Figure 3B) likely reflects enhanced cross-linking involving EWP’s cysteine residues, which become exposed during alkaline unfolding and are subsequently oxidized during ultrasonication. This compositional difference explains why US-P-pH produced the hardest gels (1020.63 g, Table 2), whereas treatments that failed to expose or crosslink these residues (e.g., US-A-pH under acidic conditions) yielded much weaker gels.

#### 2.2.4. Electrophoretic Profiles

**Figure 4:**
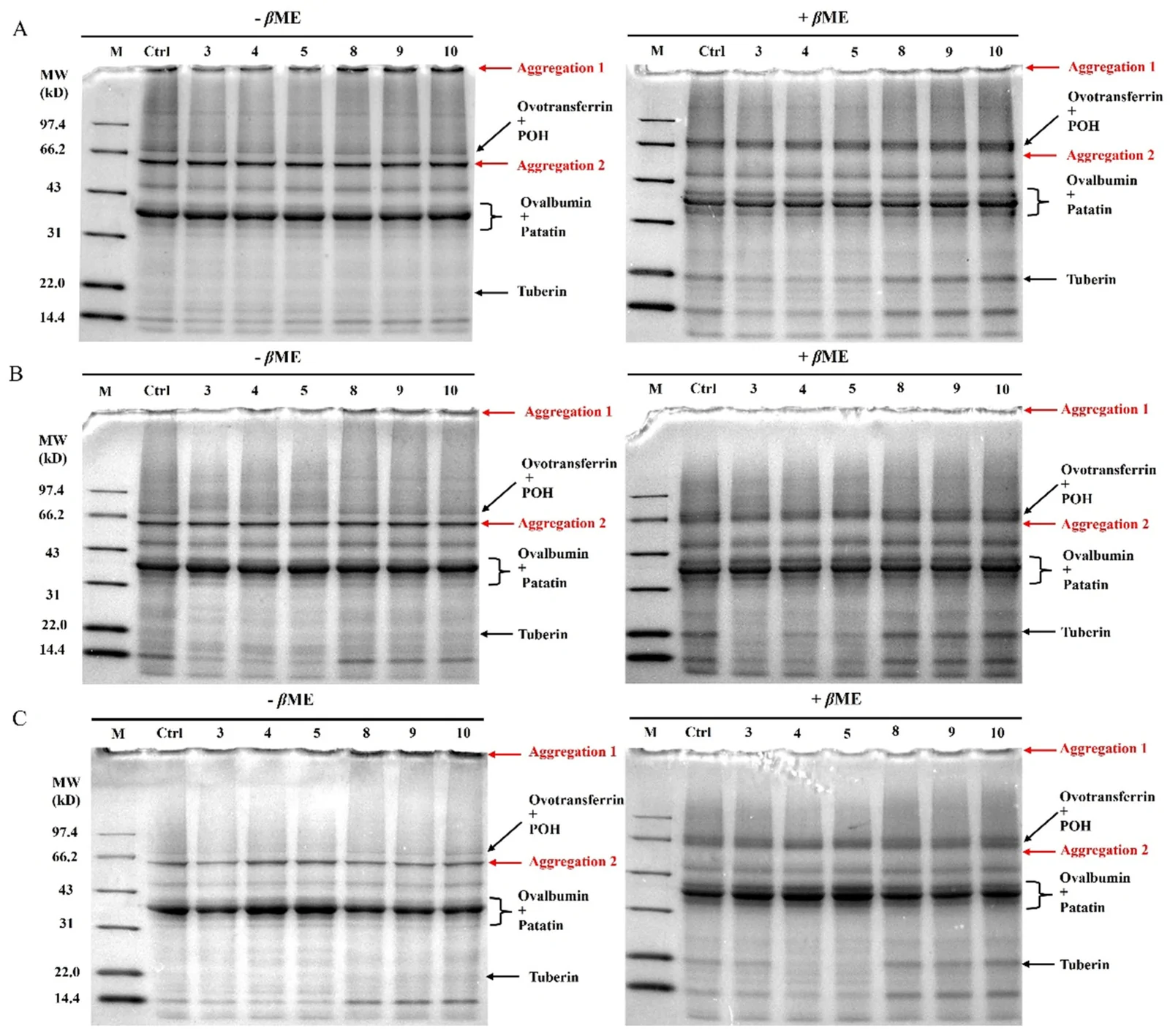
SDS–PAGE patterns of PP/EWP prepared with ultrasound before pH-shift treatment ((**A**), US-B-pH), ultrasound after pH-shift treatment ((**B**), US-A-pH), and ultrasound in the process of pH-shift treatment ((**C**), US-P-pH) under reducing (+*β*ME) and nonreducing (−*β*ME) conditions. M: marker; Ctrl: control group; 3–10: different samples prepared at the corresponding pH-shift conditions; POH: polymer of high-molecular-weight protein in PP.

To assess the impact of disulfide bonds on protein aggregation and cross-linking, SDS-PAGE analysis was conducted on the different PP/EWP samples under reducing and nonreducing conditions (Figure 4). The bands corresponding to potato proteins, such as tuberin (~22 kDa), patatin (~39–45 kDa), and polymer of high-molecular-weight protein (POH) (~75 kDa), are related to protein aggregation [28]. The EWP bands mainly corresponded to ovotransferrin (~76 kDa) and ovalbumin (~35 kDa) [29]. The results under nonreducing conditions (−*β*ME) showed that the aggregation 1 band in the alkaline and control groups was thicker than that in the acid groups, while no clear changes in this band were observed between the control group and alkaline treatments. In addition, for all the samples, a clear aggregation 2 band was observed, which indicated that aggregation 2 was induced by ultrasound rather than by the folding–refolding process. Under reducing conditions (+*β*ME), aggregations 1 and 2 were visibly degraded to ovotransferrin, POH, and tuberin. Moreover, distinct bands corresponding to ovalbumin and patatin were obtained for all the samples. Notably, after the addition of βME, aggregation 1 band was still visible for the samples treated with alkaline conditions, while aggregation 2 band completely disappeared. Thus, these results suggested that aggregates 1 and 2 were formed mainly by ovotransferrin, ovalbumin, patatin, POP, and tuberin through disulfide bonds, with minor involvement of other non-disulfide covalent bonds.

### 2.3. Effects of Modification Treatments on the Gel Properties of PP/EWP

#### 2.3.1. Texture Profile Analysis (TPA)

The texture characteristics of the PP/EWP gels under different treatments exhibited significant differences, as presented in Table 2. Overall, the mixed protein gels formed under alkaline pH-shift treatments showed superior textural properties compared to those formed under acidic pH shifts. As shown in Table 2, the hardness of gels treated with alkaline ultrasound-assisted pH-shift treatments reached 573.85 g, 481.23 g, and 221.49 g, whereas the maximum hardness values under acidic conditions were 289.61 g, 435.70 g, and 164.59 g, respectively. In addition, the adhesiveness and chewiness of the mixed protein gels formed under alkaline pH-shift treatments were also superior, especially under US-P-pH and US-A-pH protocols. The US-P-pH _pH=9_ gels exhibited the highest gel hardness (1020.63 g), adhesiveness (437.88), and chewiness (388.64), although springiness, cohesiveness, and resilience declined. This phenomenon may be related to the decreased random coil contents, increased S-S contents, higher H_0_ and lower particle size. Elasticity indicates the ability of a gel to return to its original shape after removal of the deformation force. The gel elasticity of all mixed proteins under US-B-pH treatment was better than that of the control group, indicating that flexible cross-linking of the mixed protein was enhanced after heating, forming a gel network structure capable of supporting large deformations [30]. The exceptionally high hardness of US-P-pH _pH=9_ gel (1020.63 g) was attributed to the synergistic combination of EWP’s disulfide-mediated cross-linking and PP’s patatin-induced water binding. In contrast, the high elasticity of US-B-pH gels reflected a more flexible network dominated by non-covalent interactions, likely due to ultrasound-induced fragmentation of rigid EWP aggregates.

#### 2.3.2. Water-Holding Capacity (WHC)

**Figure 5:**
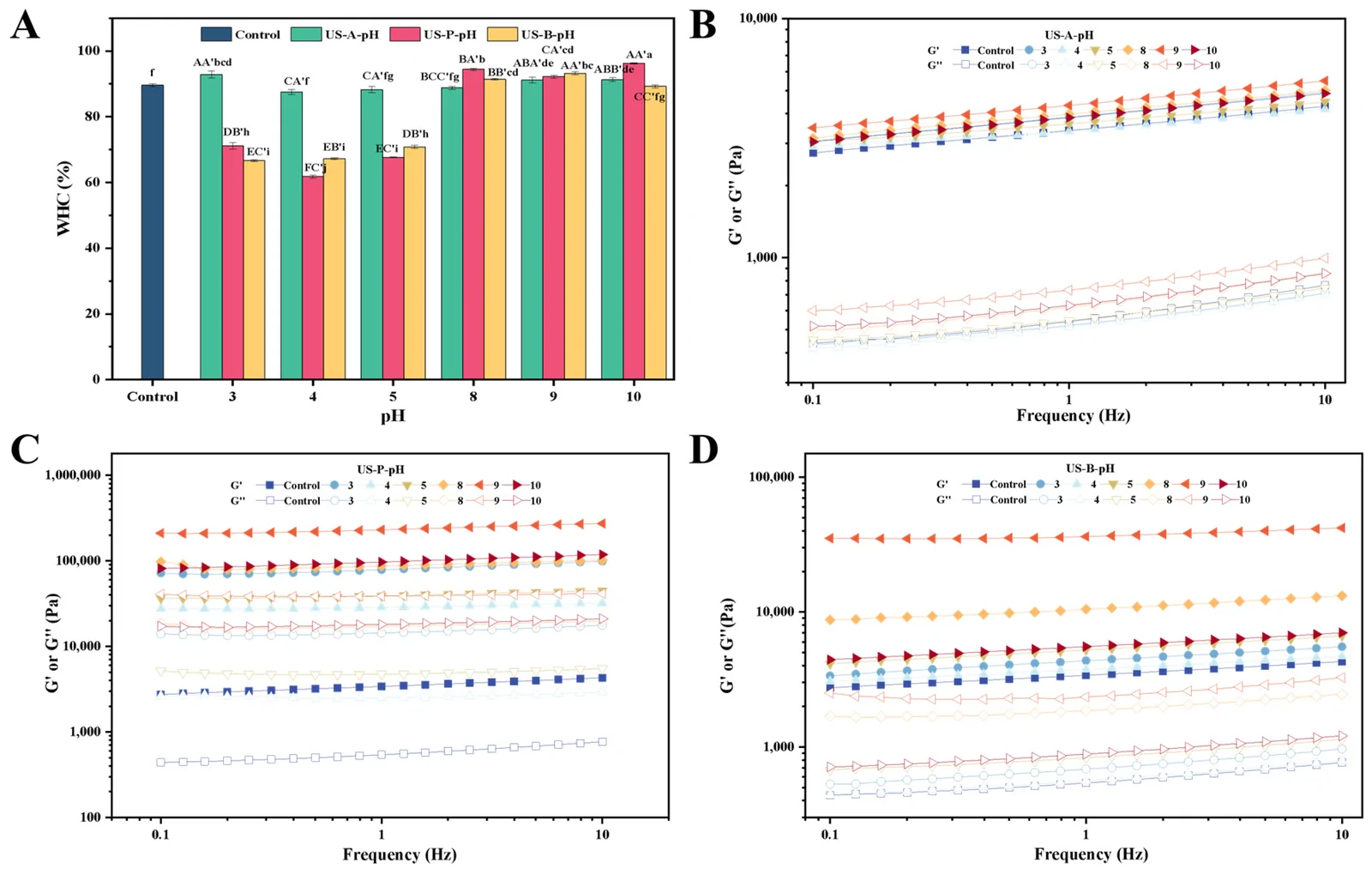
Water-holding capacity of PP/WEP gels obtained under various ultrasound and pH-shift treatments (**A**), frequency sweep plots of the storage and loss moduli for PP/WEP under ultrasound after pH-shift treatment ((**B**), US-A-pH), ultrasound in the process of pH-shift treatment ((**C**), US-P-pH), and ultrasound before pH-shift treatment ((**D**), US-B-pH). Different superscript letters indicate significant differences (*p* < 0.05).

The WHC is an important parameter for evaluating the quality of thermotropic protein gels, as it reflects the water retention ability of the gel network. Gels with higher WHC are generally more suitable for food-related applications. As shown in Figure 5A, under US-P-pH and US-B-pH conditions, the WHC of samples treated by alkaline treatments were superior to those obtained with acid pH shifts. This could be due to improved flexibility of the protein structure after alkali treatment, resulting in more exposure of polar residues. Li found that acidification caused poor gel performance, thereby reducing the WHC [31]. Oujifard reported that higher WHC was consistent with an increase in gel hardness and breaking deformation, consistent with the present results [32]. Gels with a densely distributed network structure typically have better WHC, as small and uniform pores enable more water to be trapped within the network structure. The US-P-pH _pH=10_ and US-B-pH _pH=9_ gels exhibited the maximum WHC values of 96.26% and 93.22%, respectively. This indicated that ultrasonic treatment increased the cross-linking between proteins in the composite gel, thereby enhancing the density of the network structure and thus improving the water-holding capacity.

#### 2.3.3. Rheological Analysis

The rheological behavior of the material has a significant impact on its processability, and it can be used to elucidate the interactions between the components in gel-forming food products. Frequency sweep plots provide information about the gel networks formed by covalent or non-covalent bonds [33]. As shown in Figure 5B–D, the G′ values (storage modulus) of all samples remained higher than the G″ values (loss modulus) within the frequency range of 0.1–10 Hz, indicating that the solid-state elastic properties played a dominant role, and the network was mainly maintained by non-covalent interactions [34]. The G′ values of US-P-pH were relatively low in the frequency-dependent region, suggesting that non-covalent bonds including hydrophobic interactions and hydrogen bonds may control the gel network. While the US-A-pH treatment increased the frequency dependence of the mixed protein gel, the changes in G′ and G′ of the mixed proteins after each pH treatment were not significant. This is attributed to the changes in the tertiary structure of the mixed proteins discussed above.

The above phenomenon can be explained by the power law equation models. K′ and K″ are the storage modulus and viscous modulus of the gel at 1 rad/s [35]. As presented in Table 3, under US-P-pH and US-B-pH treatments, the K′ values were higher than K″, and both constants were significantly higher compared to the control group, indicating that the gels exhibited stronger elasticity rather than viscosity. There were no significant differences in the K′ and K″ parameters between US-A-pH treatment groups and the control group, except for US-A-pH _pH=9_. This indicated that US-A-pH resulted in a greater number of loose protein aggregates in the gel than other samples. The parameters *n*′ and *n*″ reflect the frequency dependence of the modulus and the structure of gels. Generally, a low *n*′ value (close to zero) indicates that the gel behaves as a covalent gel with low frequency dependence and strong viscoelasticity [36]. The *n*′ index of US-P-pH samples was lower than that of the US-A-pH and US-B-pH samples under the same pH shifts, confirming that US-P-pH had better structural stability. For the US-A-pH and US-B-pH samples, *n*″ was higher than *n*′, which indicated that the decrease rate of G″ was greater than that of G′, and the elasticity of the gel network increased slightly as *ω* decreased [37].

#### 2.3.4. CLSM

**Figure 6:**
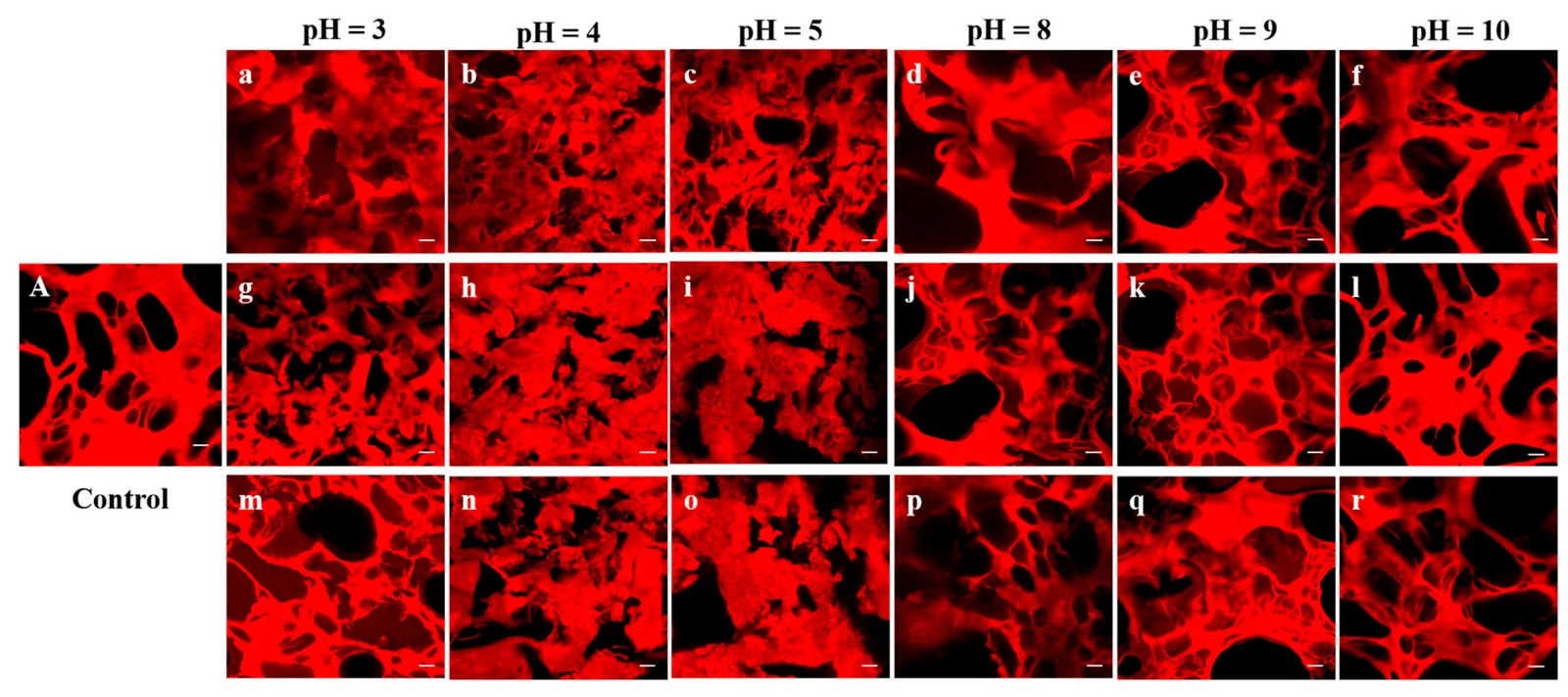
Laser scanning confocal microscopy (CLSM) images of PP/EWP gels prepared by various ultrasound and pH-shift treatments. The red color corresponds to the stained protein. The scale bars in the images correspond to an actual length of 20 μm. (**A**): PP/EWP gel without treatment; (**a**–**f**): corresponding to PP/EWP gels prepared by US-A-pH (ultrasound after pH-shift treatment); (**g**–**l**): corresponding to PP/EWP gels prepared by US-P-pH (ultrasound in the process of pH-shift treatment); (**m**–**r**): corresponding to PP/EWP gels prepared by US-B-pH (ultrasound before pH-shift treatment). PP: potato protein; EWP: egg white protein.

The CLSM images of PP/EWP gels prepared by different treatments are presented in Figure 6. The surface of mixed protein gels prepared by acidic pH shifts became rough, revealing a loose, open and thick-chain network structure. In contrast, the protein gels prepared by alkaline pH shifts exhibited cross-linked fibers and a smoother three-dimensional network structure with irregular pores. This indicated that a more easily denaturable structure was formed for proteins after alkaline treatments. In particular, US-P-pH yielded the most uniform and continuous structure among the three alkaline treatment methods. Such a dense network structure with closely interlinked chains can resist external pressure and absorb more water, thereby increasing gel strength and WHC, consistent with the results of WHC and TPA. In contrast, the protein gels prepared by acidic pH-shift treatments presented a rough structure without an interconnected network. This phenomenon can be attributed to the large and bulky particles formed during the acidic denaturation process, resulting in weaker protein cross-linking.

## 3. Conclusions

This study provided the first systematic comparison of three ultrasound intervention sequences (before, during, or after pH shift) in a PP/EWP composite protein system, demonstrating that the timing of ultrasound application critically determined the final protein properties. Alkaline pH shift with concurrent ultrasound (US-P-pH) produced the most favorable structural modifications, including increased surface hydrophobicity, enhanced disulfide cross-linking, and refined secondary structure, all of which contributed to superior gel hardness, water-holding capacity, and network uniformity. These findings advanced the understanding of how molecular-level structural modifications govern macroscopic gel functionality, while also providing a mechanistic basis for designing protein modification protocols in green food processing. It should be noted, however, that the absence of single-protein controls precluded a definitive deconvolution of the individual contributions of PP and EWP; future work incorporating such controls would be valuable. Nonetheless, the composite system studied here was directly relevant to practical applications where protein blending was intended. Overall, these results lay a foundation for developing improved protein formulations and contribute to the sustainable protein processing field by demonstrating an effective physical modification approach. Further research is still needed to validate the performance of the optimized protocol in real food matrices.

## 4. Materials and Methods

### 4.1. Materials

Potato protein (PP, protein content 85.34%, dry basis) was obtained from Kaimeite Biotechnology Co., Ltd. (Beijing, China). Egg white protein (EWP, protein content 83.23%, dry basis) was purchased from Kangde Egg Industry Co., Ltd. (Nantong, China). Proteins were stored in a desiccator at room temperature (25 ± 2 °C). All other reagents and solvents used were of analytical grade and supplied by Sigma-Aldrich LLC (Shanghai, China).

### 4.2. Modification of PP/EWP Complexes

PP and EWP in a 1:1 ratio (*w*/*w*) were dispersed in distilled water and stirred continuously with a magnetic stirrer at 4 °C for 6 h to obtain a protein dispersion (2%, *w*/*v*). Then, the pH was adjusted to 7. Ultrasound treatment was conducted with a Sonics VCX800 system (Sonics & Materials, Inc., Newtown, CT, USA). The mixed protein solution was treated at 4 °C with a pulse mode (5 s on, 5 s off) at 20 kHz and 600 W for 45 min [38]. The pH-shift treatment was performed as described by Li et al. [31]. The pH range evaluated included acidic levels of pH 3, 4, and 5, an untreated control at pH 7 (no pH shift), and alkaline levels of pH 8, 9, and 10. These values were selected on the basis of preliminary experiments, which indicated that pH values below 3 or above 10 induced excessive aggregation or irreversible denaturation. The experiment design consisted of a 3 × 6 full factorial design, incorporating three ultrasound intervention sequences (US-A-pH, US-P-pH, and US-B-pH) combined with six pH treatments (3, 4, 5, 8, 9, 10), alongside a control group subjected to ultrasound only at pH 7. All treatments were carried out in triplicate, with three independent sample preparations per condition (n = 3). During the experiment, the pH of the solutions was continuously monitored to avoid any pH changes caused by protein dissolution. The samples were freeze-dried and stored in a desiccator for subsequent detection.

### 4.3. Preparation of Protein Composite Gels

A 12% (*w*/*v*) PP/EWP dispersion was prepared in distilled water to induce gel formation. Gelation was achieved by heating the dispersed solution from 25 °C to 85 °C and holding at 85 °C for 30 min. Subsequently, the gel was immediately cooled in ice water and stored at 4 °C for 12 h prior to analysis.

### 4.4. Solubility Analysis

The solubility of the protein powders in phosphate buffer (0.01 M, pH 7.0) was determined according to the method of Sha et al. [39]. Approximately 40 mg of the sample was dispersed in 20 mL of buffer and stirred magnetically for 2 h. After centrifugation at 10,000× *g* for 15 min, the protein content in the supernatant was determined using the Bicinchoninic Acid Assay (BCA) method. The solubility percentage was calculated as the ratio of the protein concentration in the supernatant to that in the total dispersion.

### 4.5. Particle Size Analysis

The protein powders were dispersed in PBS (0.01 M, pH 7.0), and the particle size distribution was analyzed by Microtrac S3500 (Microtrac Inc., Montgomeryville, PA, USA) according to the method of Lv et al. [40].

### 4.6. Surface Hydrophobicity (H_0_) Analysis

The sample dispersion (1 mg/mL in 0.01 M PBS, pH 7.0) was centrifuged at 10,000× *g* for 25 min according to the method described by Yang et al. [41]. The protein concentration in the supernatant was then determined using the Biuret method. Subsequently, the dispersion was diluted to a concentration range of 0.001–0.5 mg/mL. Next, 1 mL of the protein solution was mixed with 10 μL of 0.008 M 1-anilinonaphthalene-8-sulfonic acid (ANS) solution (Sinopharm Chemical Reagens Co., Ltd., Beijing, China) and incubated in the dark for 15 min. The fluorescence intensity was measured using an F-2500 fluorescence spectrophotometer (Hitachi, Ltd., Tokyo, Japan) with an excitation wavelength of 390 nm and an emission wavelength of 470 nm. The H_0_ index was expressed as the initial slope of the fluorescence intensity curve plotted against protein concentration.

### 4.7. Intrinsic Fluorescence Spectroscopy Analysis

Fluorescence spectra were obtained using a F-2500 fluorescence spectrophotometer (Hitachi, Tokyo, Japan) according to the method described by Ma et al. [42]. In brief, 10 mg of freeze-dried protein sample was dispersed in 20 mL of PBS (10 mM, pH 7.0), and then centrifuged at 10,000× *g* for 15 min. The resulting supernatant (3 mL) was used for the fluorescence spectrum measurement.

### 4.8. Fourier-Transform Infrared (FTIR) Spectra Analysis

Fourier-transform infrared spectroscopy (FTIR) was performed within the range of 4000–650 cm^−1^ at a resolution of 4 cm^−1^ using a Nicolet 67 FTIR spectrometer (Thermo Fisher Scientific, Inc., Madison, WI, USA) equipped with an ATR prism crystal accessory. Approximately 4 mg of each sample was placed on the ATR crystal, and spectra were collected over 16 repeated scans.

### 4.9. Free Sulfhydryl Group and Disulfide Bond Content Analysis

The content of free sulfhydryl groups (SH_F_) was determined according to the method of Yang et al. with slight modifications [43]. Briefly, 0.1 g of the sample was dissolved in 10 mL of buffer A (0.086 M Tris, 0.09 M glycine, 0.004 M EDTA, and 8 M urea, pH 8.0). The solution was then centrifuged at 8000 rpm for 10 min. The protein concentration in the supernatant was determined using the BCA method. Approximately 3 mL of the supernatant and 0.1 mL of 5,5′-dithiobis-(2-nitrobenzoic acid) (DTNB) solution (4 mg/mL, Ellman′s reagent) were mixed in the dark and stirred magnetically for 1 h. The mixture was centrifuged at 8000 rpm for 30 min, and the absorbance of the supernatant was measured at 412 nm. Buffer A was used as the blank, and all samples were measured in quintuplicate.

The total sulfhydryl group (SH_T_) content was measured before the disulfide bond content was calculated. A total of 0.1 g of sample was dissolved in 10 mL of buffer B (buffer A containing 15 mg/mL β-mercaptoethanol, pH 8.0) and incubated for 1 h. The solution was then centrifuged, and 10 mL of 12% trichloroacetic acid (TCA) was added to the supernatant to precipitate the proteins. After centrifugation at 4000× *g* for 10 min, the precipitate was collected and washed three times with 5 mL of 12% TCA. The washed precipitate was then dissolved in 10 mL of buffer A. Ellman’s reagent (200 μL) was added to the solution, and the SH_T_ content was determined using the same method as described for SH_F_. The contents of SH_F_, SH_T_, and disulfide bonds were calculated as follows:

SH content (μmol/g) = [(A_412_ − A_0_) × D × 75.53]/C

SS content (μmol/g) = (SH_T_ − SH_F_)/2

where A_412_ is the absorbance of the sample at 412 nm, A_0_ is the absorbance of the blank at 412 nm, D is the dilution factor, and C is the soluble protein concentration (mg/mL).

### 4.10. Sodium Dodecyl Sulfate–Polyacrylamide Gel Electrophoresis (SDS-PAGE)

The molecular weight distribution of native and modified PP/EWP was determined using the SDS-PAGE method [29]. Freeze-dried samples were dissolved in water to prepare a 2 mg/mL solution, which was then mixed with an equal volume of loading buffer and heated in boiling water for 10 min. Subsequently, 20 μL of each sample was loaded into the gel wells. Electrophoresis was performed using a precast gel consisting of a 5% acrylamide stacking gel and a 12% separating gel, run at a constant voltage of 100 V for 1 h (Bio-Rad Mini-PROTEAN II/3/Tetra System, Bio-Rad Laboratories, California, USA). The gel was stained using a solution of 25% (*v*/*v*) ethanol and 8% (*v*/*v*) acetic acid containing 0.25% (*w*/*v*) Coomassie Brilliant Blue R-250, and then destained with the same solvent without the dye. Gel image analysis was performed using the Tanon 5200 Series Fully Automatic Chemiluminescence Image Analysis System (Shanghai Tianneng Life Science Co., Ltd., Shanghai, China).

### 4.11. Texture Profile Analysis

The textural properties of the gel samples were measured using a CT3 texture analyzer (Brookfield Engineering Laboratories, Inc., Middleboro, MA, USA) equipped with a P/0.5 R probe, and the TPA mode was selected for analysis [44]. The samples were cut into cylindrical shape with 12 mm diameter and 23 mm height. The operating conditions were set as follows: compression strain 50%; pre-test and post-test speeds 2 mm/s; and test speed 0.5 mm/s. Hardness, springiness, cohesiveness, resilience, gumminess, and chewiness were recorded. Each sample was tested six times.

### 4.12. Water-Holding Capacity (WHC) Analysis

The water-holding capacity (WHC) was determined according to the method described by Zhuang et al. with modifications [45]. Approximately 1.5 g of gel was wrapped in filter paper and then centrifuged at 10,000× *g* for 10 min. Excess water on the surface of the PP/EWP gel was removed by absorption with filter paper. The WHC was calculated using the following formula:

WHC (%) = [1 − [(m_1_ − m_2_)/m_0_]] × 100%

where m_1_ and m_2_ are the total weights (sample and centrifuge tube) before and after centrifugation, respectively, and m_0_ is the weight of the sample.

### 4.13. Rheological Properties

The PP/EWP powder was dispersed in distilled water under magnetic stirring for 6 h. The final sample concentration was adjusted to 12% (*w*/*v*). Dynamic rheological properties were measured using a rheometer (Physica MCR 301, Anton Paar, Graz, Austria) equipped with a 50 mm parallel plate geometry and a 1 mm gap. Before performing the frequency scan, a strain sweep was conducted to determine the linear viscoelastic (LVE) region. A layer of silicone oil was applied to the sample surface to prevent evaporation.

The gelation and melting properties of protein samples were determined through temperature sweep tests at a constant strain of 0.5% and a fixed frequency of 1 Hz, with all parameters maintained within the LVE range. The temperature sweep procedure was set to heat from 25 °C to 85 °C, hold for 30 min, and then cool back to 25 °C at a heating/cooling rate of 5 °C/min. The storage modulus (G′) and loss modulus (G″) were recorded as functions of temperature.

A dynamic frequency sweep was performed under a constant strain amplitude (γ = 0.5%). The variations in G′ and G″ were determined over a frequency range of 0.1 to 10 Hz at 25 °C.

### 4.14. Microstructure Observation

The microstructural network was observed using a confocal laser scanning microscope (CLSM) (Carl Zeiss AG, Oberkochen, Germany). The gel samples were cut to a thickness of 1 mm and then stained with 1% (*w*/*v*) rhodamine B (dissolved in dimethyl sulfoxide) for 30 min, followed by rinsing with distilled water to remove excess dye. The stained samples were placed on a microscope slide and observed at an excitation wavelength of 582 nm and a magnification of 20×.

### 4.15. Statistical Analysis

All experiments were conducted at least three times. Two-way analysis of variance (ANOVA) was performed using IBM SPSS Statistical Analysis software (version 22.0), and differences were considered significant at *p* < 0.05 according to Duncan’s test.

## Figures and Tables

**Table 1:** Secondary structure of PP/EWP after various ultrasound and pH-shift treatments.

	pH	*α*-Helix	*β*-Sheet	*β*-Turn	Random Coil
Control	7	27.88 ± 0.16 ^a^	40.20 ± 0.18 ^i^	17.07 ± 0.10 ^i^	14.85 ± 0.12 ^gh^
US-A-pH	3	18.77 ± 0.59 ^AB′fg^	38.35 ± 0.68 ^BB′a^	21.01 ± 1.68 ^CA′B′fg^	21.87 ± 1.60 ^BA′efgh^
	4	7.59 ± 0.70 ^FC′m^	36.94 ± 0.35 ^BCA′de^	28.70 ± 0.16 ^AA′a^	26.78 ± 0.31 ^AA′a^
	5	13.52 ± 0.18 ^DC′j^	43.76 ± 1.12 ^AA′a^	22.42 ± 0.19 ^BB′ef^	20.29 ± 1.07 ^ABA′cdef^
	8	11.23 ± 0.69 ^EB′k^	43.92 ± 1.01 ^AA′a^	22.19 ± 0.36 ^BCB′ef^	22.67 ± 0.59 ^ABA′bc^
	9	14.81 ± 0.47 ^CB′i^	42.39 ± 0.70 ^AA′b^	22.60 ± 0.38 ^BB′e^	20.20 ± 0.15 ^ABB′cdef^
	10	16.96 ± 0.79 ^BB′h^	35.91 ± 1.16 ^CB′ef^	22.23 ± 0.07 ^BCA′ef^	24.90 ± 2.03 ^AA′ab^
US-P-pH	3	26.39 ± 1.75 ^AA′b^	35.69 ± 0.53 ^BC′ef^	19.02 ± 1.97 ^DB′h^	18.90 ± 0.11 ^BA′cdefg^
	4	22.39 ± 0.80 ^CA′d^	35.10 ± 0.53 ^BCB′fg^	22.97 ± 0.76 ^CC′e^	19.54 ± 0.75 ^BB′cdef^
	5	24.50 ± 0.46 ^BA′c^	33.60 ± 0.60 ^DC′h^	24.89 ± 1.13 ^BA′d^	17.01 ± 0.39 ^CB′fgh^
	8	20.82 ± 0.26 ^CA′e^	34.23 ± 0.17 ^CDC′gh^	27.48 ± 0.33 ^AA′ab^	17.47 ± 0.10 ^CC′efgh^
	9	21.85 ± 0.65 ^CA′de^	38.38 ± 1.22 ^AB′a^	26.27 ± 0.42 ^ABA′bc^	13.51 ± 1.00 ^DC′h^
	10	21.93 ± 0.45 ^CA′de^	33.52 ± 0.12 ^DC′h^	22.71 ± 0.43 ^CA′e^	21.84 ± 0.64 ^AB′bcd^
US-B-pH	3	17.85 ± 0.33 ^CB′gh^	41.16 ± 1.14 ^AA′b^	22.81 ± 0.41 ^BA′e^	18.18 ± 0.68 ^DA′defg^
	4	19.18 ± 0.47 ^BB′f^	36.63 ± 0.70 ^BA′de^	25.28 ± 1.22 ^AB′cd^	18.91 ± 0.16 ^DB′cdefg^
	5	19.66 ± 0.65 ^BB′f^	37.62 ± 1.07 ^BB′ab^	21.10 ± 0.33 ^CB′fg^	21.62 ± 0.59 ^BA′bcde^
	8	21.30 ± 0.60 ^AA′de^	37.40 ± 0.59 ^BB′ab^	20.77 ± 0.42 ^CC′g^	20.53 ± 1.44 ^BCB′cdef^
	9	10.11 ± 0.45 ^DC′l^	41.29 ± 0.55 ^AA′b^	20.05 ± 0.23 ^CC′gh^	28.54 ± 1.10 ^AA′a^
	10	21.06 ± 0.47 ^AA′e^	37.47 ± 0.05 ^BA′ab^	22.21 ± 0.43 ^BA′ef^	19.26 ± 0.29 ^CDC′cdef^

US-A-pH: ultrasound after pH-shift treatment; US-P-pH: ultrasound in the process of pH-shift treatment; US-B-pH: ultrasound before pH-shift treatment; Different superscript letters indicate significant differences (*p* < 0.05).

**Table 2:** Impact of various ultrasound and pH-shift treatments on the texture profile analysis (TPA) parameters of PP/EWP gels.

	pH	Hardness/g	Springiness/%	Cohesiveness	Resilience/%	Gumminess	Chewiness
Control	7	467.58 ± 8.95 ^e^	92.93 ± 2.58 ^efg^	0.48 ± 0.02 ^ab^	10.33 ± 0.66 ^gf^	149.63 ± 5.56 ^f^	135.26 ± 5.35 ^g^
US-A-pH	3	405.08 ± 20.34 ^DA′g^	91.91 ± 2.62 ^BCB′fgh^	0.46 ± 0.01 ^BCA′bc^	12.88 ± 0.33 ^AA′bc^	185.34 ± 8.57 ^BCA′de^	170.09 ± 6.39 ^BA′ef^
	4	401.16 ± 12.48 ^DA′g^	87.55 ± 1.05 ^DB′ij^	0.44 ± 0.01 ^CA′cd^	10.47 ± 0.12 ^CA′fg^	177.79 ± 4.14 ^CA′e^	155.59 ± 2.39 ^CA′fg^
	5	435.70 ± 12.34 ^CA′f^	91.72 ± 0.93 ^BCB′fgh^	0.44 ± 0.01 ^CC′cd^	11.03 ± 0.59 ^BCA′ef^	192.26 ± 8.27 ^BA′d^	176.45 ± 8.97 ^BA′e^
	8	481.23 ± 20.86 ^BB′e^	97.06 ± 0.28 ^AB′cde^	0.48 ± 0.02 ^ABA′ab^	10.57 ± 0.70 ^BCB′fg^	231.62 ± 6.86 ^AB′c^	224.77 ± 6.02 ^AB′d^
	9	484.10 ± 9.77 ^BB′e^	94.98 ± 1.31 ^ABA′def^	0.49 ± 0.01 ^AA′a^	12.25 ± 0.51 ^AA′cd^	235.44 ± 4.27 ^AB′c^	223.54 ± 3.07 ^AB′d^
	10	524.28 ± 2.93 ^AB′d^	90.24 ± 3.27 ^CDB′ghi^	0.45 ± 0.00 ^CA′B′c^	11.42 ± 0.17 ^BB′e^	236.20 ± 1.72 ^AB′c^	213.27 ± 9.33 ^AB′d^
US-P-pH	3	289.61 ± 5.46 ^DB′ij^	60.05 ± 0.52 ^DC′k^	0.37 ± 0.02 ^CC′f^	4.49 ± 0.17 ^DC′l^	115.91 ± 3.99 ^CB′h^	69.67 ± 2.48 ^DB′i^
	4	271.98 ± 8.11 ^DB′j^	40.29 ± 1.70 ^EC′l^	0.28 ± 0.01 ^DB′g^	3.47 ± 0.08 ^EC′m^	75.69 ± 0.58 ^DB′j^	30.67 ± 1.32 ^EC′j^
	5	276.07 ± 3.46 ^DB′j^	85.36 ± 1.59 ^CC′j^	0.49 ± 0.01 ^AA′a^	4.81 ± 0.10 ^DB′kl^	133.24 ± 2.72 ^CB′g^	112.63 ± 3.28 ^CB′h^
	8	573.85 ± 16.57 ^CA′c^	114.18 ± 5.36 ^AA′a^	0.45 ± 0.01 ^BB′c^	13.08 ± 0.26 ^BA′b^	281.06 ± 7.81 ^BA′b^	280.06 ± 10.28 ^BA′c^
	9	1020.63 ± 19.23 ^AA′a^	88.70 ± 4.11 ^CB′hij^	0.39 ± 0.01 ^CC′e^	9.14 ± 1.06 ^CB′h^	437.88 ± 24.56 ^AA′a^	388.64 ± 42.19 ^AA′a^
	10	663.50 ± 11.07 ^BA′b^	101.61 ± 3.10 ^BA′b^	0.44 ± 0.01 ^BC′cd^	14.51 ± 0.12 ^AA′a^	289.20 ± 10.57 ^BA′b^	318.80 ± 18.57 ^BA′b^
US-B-pH	3	164.59 ± 5.02 ^DC′l^	97.66 ± 0.45 ^ABA′cde^	0.40 ± 0.01 ^CB′e^	6.12 ± 0.34 ^DB′i^	65.65 ± 1.25 ^EA′j^	64.12 ± 1.32 ^DB′i^
	4	149.89 ± 3.44 ^EC′l^	98.21 ± 0.12 ^ABA′bcd^	0.42 ± 0.02 ^CA′d^	5.55 ± 0.19 ^DEB′ij^	63.12 ± 2.50 ^EC′j^	61.98 ± 2.41 ^DB′i^
	5	163.49 ± 6.07 ^DC′l^	96.87 ± 1.39 ^BCA′cde^	0.46 ± 0.01 ^ABB′bc^	5.36 ± 0.13 ^EB′jk^	75.59 ± 2.62 ^DC′j^	73.23 ± 2.84 ^CC′i^
	8	306.86 ± 10.33 ^BC′i^	99.30 ± 0.26 ^AA′bc^	0.48 ± 0.01 ^AA′B′ab^	10.14 ± 0.19 ^BB′A′g^	143.47 ± 4.93 ^BC′fg^	142.49 ± 5.19 ^AC′g^
	9	340.99 ± 8.50 ^AC′h^	95.55 ± 1.43 ^CDB′cdef^	0.45 ± 0.00 ^BA′c^	11.73 ± 0.60 ^Ade^	153.45 ± 4.37 ^AC′f^	146.46 ± 2.55 ^AC′g^
	10	221.499.43 ^CC′k^	94.71 ± 1.30 ^DB′def^	0.46 ± 0.01B ^bc^	9.13 ± 0.18 ^CC′h^	100.59 ± 3.93 ^CC′i^	95.21 ± 3.32BC ^′^^h^

US-A-pH: ultrasound after pH-shift treatment; US-P-pH: ultrasound in the process of pH-shift treatment; US-B-pH: ultrasound before pH-shift treatment; Different superscript letters indicate significant differences (*p* < 0.05).

**Table 3:** Fitting parameters of frequency sweep spectra of PP/EWP gels with various ultrasound and pH-shift treatments.

	pH	$G^{\prime }=K^{\prime }\omega^{n^{\prime }}$			$G=K^{\prime \prime }\omega^{n^{\prime \prime }}$		
		*K′ *(Pa)	*n′*	*R^2^*	*K″* (Pa)	*n″*	*R^2^*
Control	7	3413.03 ± 3.29 ^k^	0.096 ± 0.000 ^cd^	0.999	554.36 ± 2.99 ^L^	0.127 ± 0.004 ^abc^	0.983
US-A-pH	3	3876.18 ± 2.06 ^CC′jk^	0.102 ± 0.000 ^AA′ab^	0.999	534.14 ± 2.73 ^EC′l^	0.110 ± 0.004 ^DB′f^	0.979
	4	3436.16 ± 9.44 ^FC′k^	0.078 ± 0.002 ^EB′f^	0.988	521.71 ± 2.05 ^FC′l^	0.12 ± 0.003 ^ABB′cde^	0.989
	5	3650.25 ± 6.68 ^EC′k^	0.084± 0.001 ^DB′e^	0.995	556.29 ± 2.56 ^DC′l^	0.116 ± 0.003 ^BCA′def^	0.984
	8	3981.69 ± 2.37 ^BC′jk^	0.098± 0.000 ^CA′bc^	0.999	629.92 ± 2.26 ^CC′kl^	0.123 ± 0.002 ^AA′bcd^	0.991
	9	4348.25 ± 3.97 ^AC′j^	0.098± 0.000 ^CA′bc^	0.999	745.63 ± 3.09 ^AC′jk^	0.114 ± 0.003 ^CDB′ef^	0.987
	10	3850.50 ± 1.17 ^DC′jk^	0.100 ± 0.000 ^BA′bc^	0.999	641.62 ± 2.58 ^BC′kl^	0.117 ± 0.003 ^BCA′def^	0.988
US-P-pH	3	80,060.46 ± 401.51 ^DA′d^	0.083 ± 0.004 ^AB′e^	0.964	14,750.44 ± 101.13 ^DA′d^	0.061 ± 0.005 ^AC′i^	0.883
	4	28,964.24 ± 91.33 ^EA′g^	0.036 ± 0.002 ^DC′j^	0.924	2631.11 ± 23.77 ^FA′f^	0.018 ± 0.006 ^DEC′lm^	0.805
	5	39,436.44 ± 105.47 ^FA′e^	0.049 ± 0.002 ^CC′h^	0.970	4926.20 ± 43.75 ^DA′e^	0.022 ± 0.006 ^DB′l^	0.905
	8	89,236.77 ± 1125.84 ^CA′c^	0.042 ± 0.009 ^CB′i^	0.915	17,596.50 ± 166.03 ^CA′c^	0.036 ± 0.007 ^CC′k^	0.936
	9	232,911.96 ± 708.53 ^AA′a^	0.064 ± 0.002 ^BB′g^	0.978	39,375.58 ± 185.74 ^AA′a^	0.012 ± 0.003 ^EA′m^	0.868
	10	97,105.01 ± 109.81 ^BA′b^	0.083 ± 0.000 ^AC′e^	0.998	18,253.34± 71.70 ^BA′b^	0.051 ± 0.003 ^BB′j^	0.942
US-B-pH	3	4344.37 ± 3.63 ^EB′j^	0.105 ± 0.000 ^AA′a^	0.999	693.70 ± 2.33 ^EB′k^	0.134 ± 0.002 ^AA′a^	0.994
	4	3772.85 ± 3.01 ^FB′jk^	0.092 ± 0.000 ^DA′d^	0.999	570.35 ± 2.25 ^FB′l^	0.129 ± 0.003 ^AA′ab^	0.991
	5	5235.69 ± 2.75 ^DB′i^	0.102 ± 0.000 ^BA′ab^	0.999	848.49 ± 3.72 ^DB′ij^	0.117 ± 0.003 ^BA′def^	0.986
	8	10,565.78 ± 23.58 ^BB′h^	0.092 ± 0.002 ^DA′d^	0.994	1915.66 ± 13.27 ^BB′h^	0.092 ± 0.005 ^CB′g^	0.946
	9	37,106.28 ± 168.97 ^AB′f^	0.068 ± 0.003 ^CA′bc^	0.892	2520.77 ± 36.98 ^AB′g^	0.075 ± 0.010 ^DC′h^	0.707
	10	5555.21 ± 3.27 ^CB′i^	0.099 ± 0.000 ^CB′bc^	0.999	898.93 ± 3.12 ^CB′i^	0.120 ± 0.002 ^BA′cde^	0.992

US-A-pH: ultrasound after pH-shift treatment; US-P-pH: ultrasound in the process of pH-shift treatment; US-B-pH: ultrasound before pH-shift treatment; Different superscript letters indicate significant differences (*p* < 0.05).

## Data Availability

The original contributions presented in this study are included in the article/Supplementary Material. Further inquiries can be directed to the corresponding author(s).

## Supplementary Materials

The following supporting information can be downloaded at: https://www.mdpi.com/article/10.3390/gels12080686/s1, Figure S1:The SDS–PAGE patterns of material proteins. M: marker, PP: potato protein, EWP: egg white protein.; Figure S2: Size distribution results of PP/EWP prepared from ultrasound after pH-shift treatment (US-A-pH), ultrasound in the process of pH-shift treatment (US-P-pH), and ultrasound before pH-shift treatment (US-B-pH).
