# Supplementary material for: Differential Effects of pH-Shift and Ultrasound Intervention Sequences on Physicochemical, Structural and Gelling Properties of Potato–Egg White Composite Proteins
Source: Gels. 2026 Aug 3;12(8):686. doi: 10.3390/gels12080686 (PMC13512436; doi:10.3390/gels12080686)
Supplement: Supplementary file 1 [file gels-12-00686-s001.zip › gels-4367919-supplementary.pdf]

Effects of pH-shift processing assisted by ultrasonication on physicochemical, structural and gelling properties of potato–egg white composited proteins

Jing Li [a, 1], Huimin Lu [a, b, 1], Hongxi Zhao [a, c], Qiannan Liu [a], Ruixuan Zhao [a, *], Honghai Hu[a, *]

[a] Institute of Food Science and Technology, Chinese Academy of Agricultural Sciences, Comprehensive Key Laboratory of Agro-products Processing, Ministry of Agriculture and Rural Affairs, Beijing 100193, P.R. China.

[b] Guangdong Provincial Key Laboratory of Aquatic Product Processing and Safety, College of Food Science and Technology, Guangdong Ocean University, Zhanjiang, Guangdong, 524088, P.R. China.

[c] State Key Laboratory of Food Nutrition and Safety, Food Biotechnology Engineering Research Center of Ministry of Education, Tianjin University of Science & Technology, Tianjin 300457, P.R. China.

[1] These authors contributed equally to this work.

*Corresponding author:

Honghai Hu, E-mail: huhonghai@caas.cn, Telephone: +86-10-62816503

Ruixuan Zhao, E-mail: zhaoruixuan@caas.cn, Telephone: +86-10-62816503

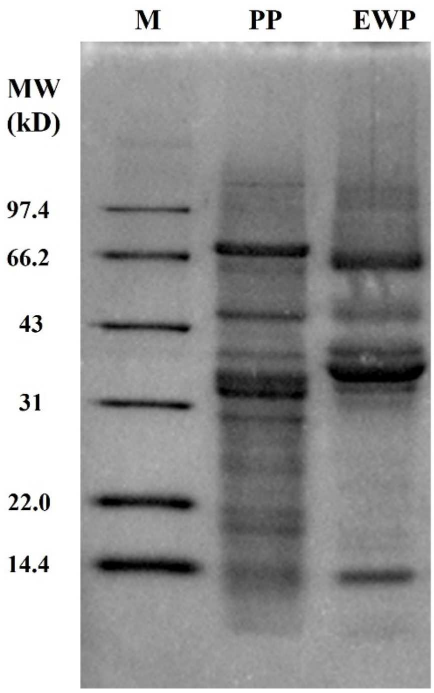

Figure S1. The SDS–PAGE patterns of material proteins. M: marker; PP: potato protein; EWP: egg white protein.

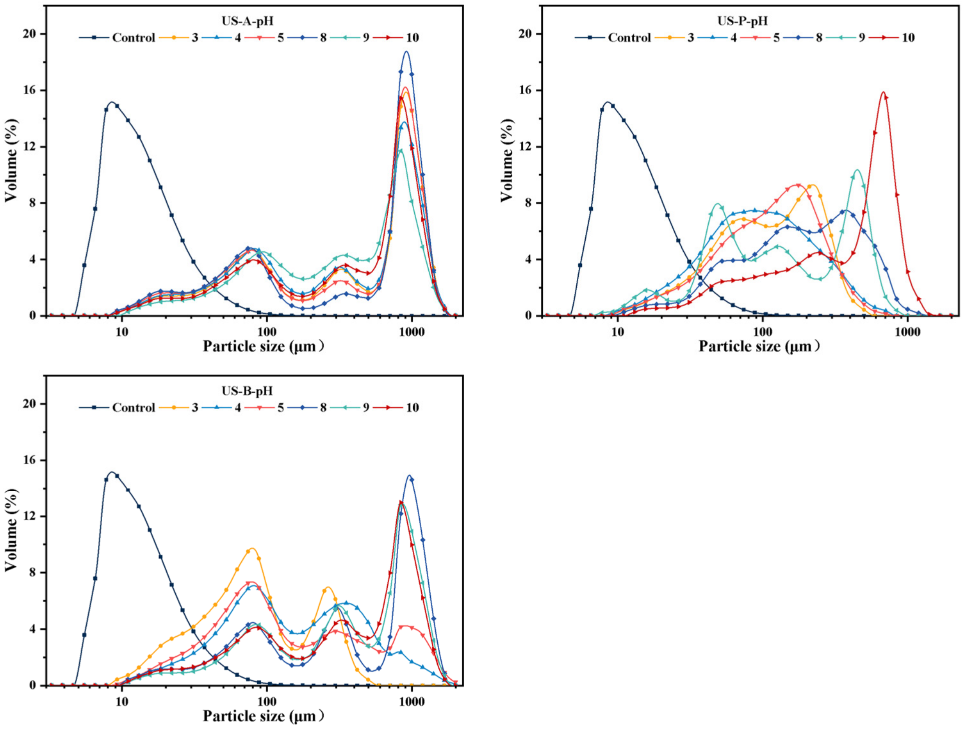

Figure S2. Size distribution results of PP/EWP prepared from ultrasound after pH-shift treatment (US-A-pH), ultrasound in the process of pH-shift treatment (US-P-pH), and ultrasound before pH-shift treatment (US-B-pH).
